# Arsenic Impairs Wound Healing Processes in Dermal Fibroblasts and Mice

**DOI:** 10.3390/ijms25042161

**Published:** 2024-02-10

**Authors:** Sara R. Dresler, Bronson I. Pinto, Matthew C. Salanga, Catherine R. Propper, Savannah R. Berry, Robert S. Kellar

**Affiliations:** 1 Department of Biological Sciences, Northern Arizona University, Flagstaff, AZ 86011, USA; srd248@nau.edu (S.R.D.); bip5@nau.edu (B.I.P.); matthew.salanga@nau.edu (M.C.S.); catherine.propper@nau.edu (C.R.P.); srb364@nau.edu (S.R.B.); 2 Center for Materials Interfaces in Research & Applications, ¡MIRA!, Flagstaff, AZ 86011, USA

**Keywords:** *MMP1*, *As3mt*, scratch assay, environmental contaminants, endocrine disruption

## Abstract

Inorganic arsenic (NaAsO_2_) is a naturally occurring metalloid found in water resources globally and in the United States at concentrations exceeding the U.S. Environmental Protection Agency Maximum Contamination Level of 10 ppb. While exposure to arsenic has been linked to cancer, cardiovascular disease, and skin lesions, the impact of arsenic exposure on wound healing is not fully understood. Cultured dermal fibroblasts exposed to NaAsO_2_ displayed reduced migration (scratch closure), proliferation, and viability with a lowest observable effect level (LOEL) of 10 µM NaAsO_2_ following 24 h exposure. An enrichment of Matrix Metalloproteinase 1 (*MMP1*) transcripts was observed at a LOEL of 1 µM NaAsO_2_ and 24 h exposure_._ In vivo, C57BL/6 mice were exposed to 10 µM NaAsO_2_ in their drinking water for eight weeks, then subjected to two full thickness dorsal wounds. Wounds were evaluated for closure after 6 days. Female mice displayed a significant reduction in wound closure and higher erythema levels, while males showed no effects. Gene expression analysis from skin excised from the wound site revealed significant enrichment in Arsenic 3-Methyltransferase (*As3mt)* and Estrogen Receptor 2 (*Esr2)* mRNA in the skin of female mice_._ These results indicate that arsenic at environmentally relevant concentrations may negatively impact wound healing processes in a sex-specific manner.

## 1. Introduction

Arsenic is a well-documented environmental human health hazard, with chronic ingestion of contaminated drinking water being the most prevalent route of exposure [1,2,3,4]. In 2000, approximately 50 million Bangladesh citizens were exposed to high levels of arsenic by way of contaminated drinking water, which was considered the largest population-level poisoning in history [5]. As a result of large-scale contaminations, such as happened in Bangladesh, the World Health Organization (WHO) and the United States Environmental Protection Agency (USEPA) established a maximum contaminant level (MCL) in drinking water of 10 µg/L (10 ppb) in 2003, which was lowered from the previous MCL of 50 µg/L [6,7].

Although the MCL was lowered in 2003, many water resources in the USA still exceed 10 ppb. In the southwest United States, numerous groundwater, well water, and springs have been documented with concerning levels of arsenic [8]. For example, in Verde Valley, AZ, Montezuma Well contains 210 µg/L (210 ppb) of arsenic [9], groundwater around the Verde River, AZ, contains 16 µg/L (16 ppb) of arsenic on average, with peak values reaching 1.3 mg/L (1.3 ppm) [10]. Notably, many wells in the Verde Valley exceed 50 µg/L (50 ppb) [11]. Similar exceedances occur across Arizona [12].

While exposure to arsenic at or below the MCL is considered safe by some authorities, studies have shown detrimental results from chronic arsenic exposure below the MCL, including prostate cancer [13], diabetes [14,15,16], cardiovascular diseases [17,18,19], and disrupted microbiomes in non-human organisms such as zebrafish [20]. A 2010 review described arsenic-induced shifts in the expression of genes responsible for numerous cellular processes, including oxidative stress, inflammation, proteotoxicity, proliferation, DNA repair, cell cycle control, and apoptosis [21]. Wound healing requires the orchestration of several of these processes and, therefore, could be at risk for disruption from arsenic exposure.

Exposure to very high concentrations of arsenic, such as those found in the Bangladesh disaster, increases the risk of dermal skin lesions, leaving chronically exposed populations at risk for subsequent co-morbidities associated with open skin wounds [22,23,24]. Moreover, arsenic impairs dermal fibroblast viability [25] and functions such as cellular migration, and proliferation, which are key components in wound closure [26,27]. These studies suggest that arsenic disrupts skin homeostasis, which may lead to poor wound healing outcomes, and places chronically exposed populations at higher risk for infections in healing wounds.

Toxic metalloids, such as arsenic, have the potential to interact with endogenous hormones and can disrupt cellular signaling. Hormonal influences, especially estrogen, are imperative to the proper healing of wounds [28]. Estrogen prohibits excessive recruitment of neutrophils to the wound site post-wound bed clearance [29] in an anti-inflammatory role that contributes to normal wound healing. Arsenic has been shown to bind to estrogen [30], potentially limiting its availability at the receptor, and low dose exposure to arsenic inhibits estrogen-regulated gene expression both in vitro and in vivo [31]. These studies suggest that arsenic exposure may inhibit the estrogenic effects on wound healing and impart a greater risk of developing chronic wounds. While the effects of estrogen on arsenic-exposed dermal fibroblasts have been described previously [26], the effects of these complex interactions have not been studied in vivo, which is imperative to understanding how wound healing can be influenced.

In the current studies, the effects of environmentally relevant concentrations of sodium arsenite (NaAsO_2_) on dermal fibroblast scratch closure, metabolic activity, viability, and gene expression were evaluated. In addition, the impacts of NaAsO_2_ exposure on wound healing components, including wound closure, wound erythema, and gene expression were investigated in vivo using a murine full-thickness wound model. These studies unmask the detriments of NaAsO_2_ on wound healing functions in both in vitro and in vivo models.

## 2. Results

### 2.1. NaAsO_2_ Exposure Impairs Scratch Closure and Upregulates MMP1 in Cultured Dermal Fibroblasts

**Figure 1:**
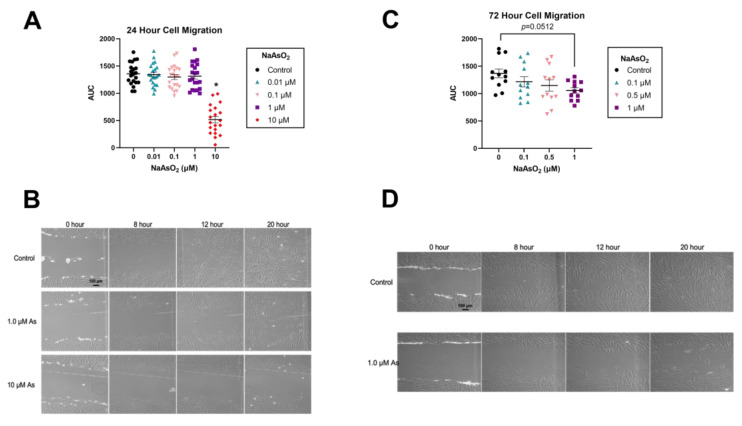
NaAsO_2_ exposure for 24 h and 72 h and its effects on scratch closure. (**A**) Quantification of dermal fibroblast migration in the scratch assay following NaAsO_2_ exposure for 24 h. Mean area under the curve (AUC) values for each treatment group are represented +/− SEM (*n* = 21, F = 53.58, *p* < 0.001). (**B**) Light micrographs of cells from the 24 h arsenic exposure scratch assay over a 20 h period. (**C**) Quantification of dermal fibroblast migration in the scratch assay following NaAsO_2_ exposure for 72 h. Mean AUC values for each treatment group are represented +/− SEM (*n* = 12, F = 2.51, *p* = 0.0512). (**D**) Light micrographs of cells from the 72 h arsenic exposure scratch assay over a 20 h period. 72 pre-scratch exposure to 10 µM arsenic killed all cells thus no images were taken. * Statistically significant values (*p* < 0.05) compared to control (Tukey’s post hoc test).

Arsenic slowed scratch closure in human dermal fibroblasts in both 24 and 72 h exposures. While a 24 h exposure to 1 µM NaAsO_2_ prior to scratching had little effect on scratch closure, 24 h exposure to 10 µM NaAsO_2_ prior to scratching significantly decreased scratch closure compared to control, and a LOEL was determined (*n* = 21, F = 53.58, *p* < 0.001; Figure 1A,B). A 72 h exposure to 1 µM NaAsO_2_ prior to scratching decreased scratch closure, although not significantly, while a 72 h exposure to 10 µM NaAsO_2_ was cytotoxic (*n* = 12, F = 2.51, *p* = 0.0512; Figure 1C,D).

**Figure 2:**
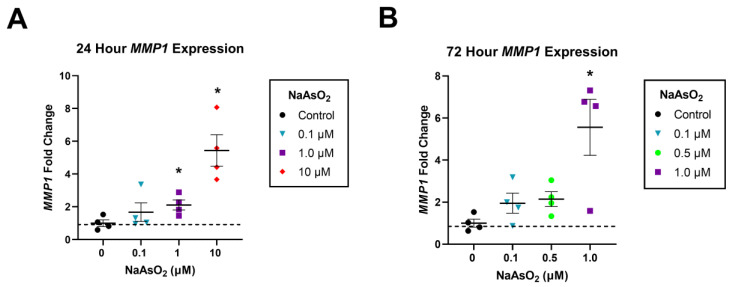
Relative gene expression of *MMP1* in dermal fibroblasts exposed to NaAsO_2_. (**A**) Effect of 0, 0.1, 1, and 10 µM NaAsO_2_ on *MMP1* gene expression in dermal fibroblasts collected after 24 h in the scratch closure assay (*n* = 4, F = 12.1, *p* < 0.001). (**B**) Effect of 0, 0.1, 0.5, and 1 µM NaAsO_2_ on *MMP1* gene expression in dermal fibroblasts collected after 72 h in the scratch closure assay (*n* = 4, F = 12.1, *p* < 0.001). Individual data points represent fold changes per replicate +/− SEM. * Statistically significant values (*p* < 0.05) compared to control (Tukey’s post hoc test).

Human dermal fibroblasts exposed to 1 and 10 µM NaAsO_2_ in the scratch assay additionally displayed an upregulation of *MMP1* expression at 24 h *(n* = 4, F = 12.1, *p* < 0.001). A 24 h exposure to 1 µM NaAsO_2_ resulted in a 2.14-fold upregulation of *MMP1* compared to control (Tukey’s, *p* < 0.05), and a 24 h exposure to 10 µM NaAsO_2_ led to a 5.4-fold upregulation of *MMP1* compared to control (ANOVA, *n* = 4, F = 12.1, *p* < 0.001; Figure 2A). A 72 h exposure to 1 µM NaAsO_2_ resulted in a 5.56-fold upregulation of *MMP1* compared to control, while a 72 h exposure to 10 µM NaAsO_2_ was cytotoxic (Tukey’s, *p* < 0.05; Figure 2B).

### 2.2. Exposure to NaAsO_2_ in Dermal Fibroblasts Alters Cell Morphology and Slows Proliferation

**Figure 3:**
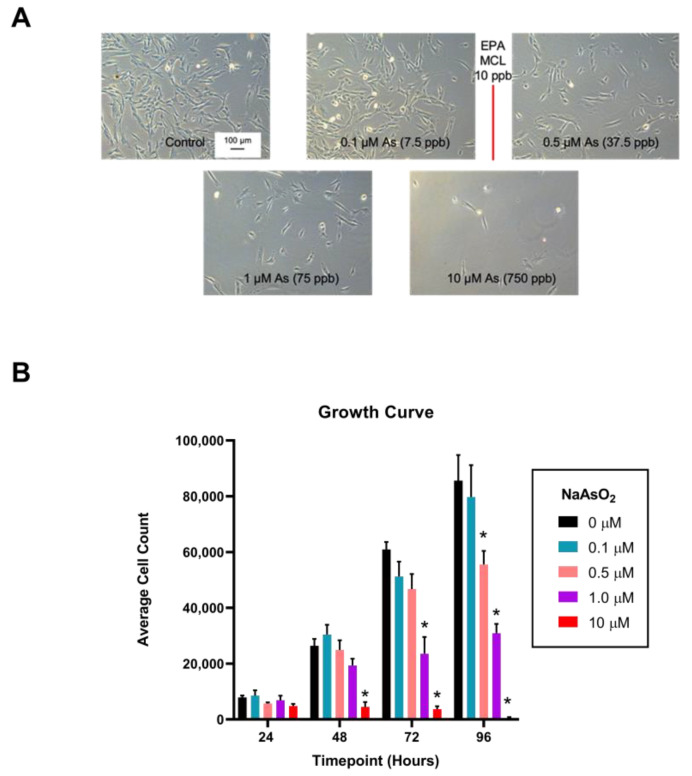
NaAsO_2_ exposure over a 96 h time period and its effects on dermal fibroblast proliferation. (**A**) Light micrographs of cells exposed to NaAsO_2_ for 96 h. (**B**) Effect of arsenic exposure on dermal fibroblast proliferation over a 96 h period. Mean cell counts are represented for each day and treatment group +/− SEM (*n* = 4). * Statistically significant values (*p* < 0.05) compared to control (Tukey’s post hoc test).

Arsenic exposure affected overall cellular appearance and growth of fibroblasts in a dose-dependent pattern. Control and 0.1 µM NaAsO_2_-exposed fibroblasts remained adherent and displayed spindle-like morphology with lamellipodial and filopodial extensions protruding outward from the cell body, indicative of a ‘normal’ cell. In contrast, the 0.5, 1, and 10 µM NaAsO_2_-treated cells appeared rounded in shape and were frequently observed suspended in the media indicating a loss of adherence (Figure 3A). Cell counting revealed lower population numbers after 48 h in the presence of 10 µM NaAsO_2_-treatment (*n* = 4, F = 12.86, *p* < 0.001), with reduced growth appearing at 72 h of exposure with 1 µM NaAsO_2_ (*n* = 4, F = 26.81, *p* < 0.05; Figure 3B). By 96 h, reduced cell growth was observed in the 0.5 µM NaAsO_2_-treatment (*n* = 4, F = 25.01, *p* < 0.01; Figure 3B). After 96 h of growth, the 0.5, 1, and 10 µM NaAsO_2_ treatments significantly slowed cellular proliferation with average cell counts of 55,527, 30,895, and 417, respectively, compared to control. After 144 h of growth, cellular proliferation contributed no further changes or return to baseline.

### 2.3. NaAsO_2_ Exposure for 24 h at the Time of Scratch Reduces Cell Metabolism and Alters DNA Quantity

**Figure 4:**
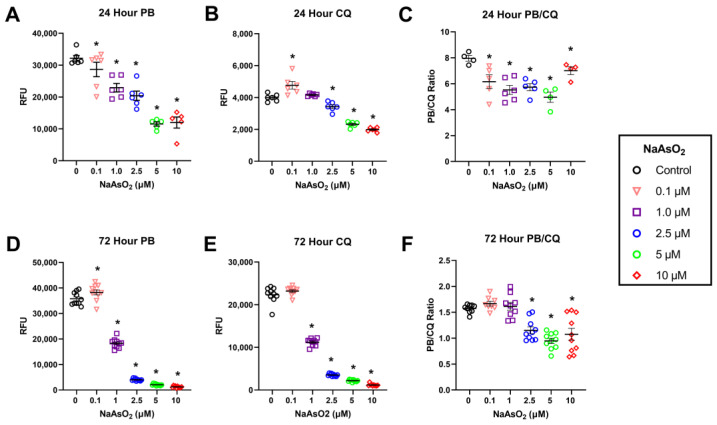
NaAsO_2_ exposure for 24 h and 72 h and its effects on metabolic activity, viability, and metabolic ratio (value of PrestBlue:CyQuant) (**A**) Effect of 24 h NaAsO_2_ exposure on metabolic activity. Mean RFU values for each treatment group are represented +/− SEM (*n* = 6, F = 29.19, *p* < 0.001). (**B**) Effect of 24 h NaAsO_2_ exposure on DNA content. Mean RFU values for each treatment group are represented +/− SEM (*n* = 6, F = 131.9, *p* < 0.001). (**C**) Effect of 24 h NaAsO_2_ exposure on metabolic output per unit of viable DNA. The ratio of RFU values for each treatment group are represented +/− SEM (*n* = 6, F = 7.58, *p* < 0.001). (**D**) Effect of 72 h NaAsO_2_ exposure on metabolic activity. Mean RFU values for each treatment group are represented +/− SEM (*n* = 10, F = 2040.7, *p* < 0.001). (**E**) Effect of 72 h NaAsO_2_ exposure on DNA content. Mean RFU values for each treatment group are represented +/− SEM (*n* = 10, F = 2735.1, *p* < 0.001). (**F**) Effect of 72 h NaAsO_2_ exposure on metabolic output per unit DNA. The ratio of RFU values for each treatment group are represented +/− SEM (*n* = 10, F = 19.6, *p* < 0.001). * Statistically significant values (*p* < 0.05) compared to control (Tukey’s post hoc test).

While PrestoBlue is an indicator of viable DNA of all the cells in any one well, which under conditions of cell stress, such as occurs during exposure to toxicants, may not be a true measure of cell viability. Therefore, we used a combination of the PrestoBlue assay and the CyQuant assay, which is used as a proliferation assay for determining metabolic activity, and the combination of PrestoBlue and CyQuant as a measure of metabolism/living DNA to represent a cell metabolic index. Fibroblasts exposed to NaAsO_2_ for 24 h displayed a dose-dependent decrease in metabolism measured by PrestoBlue as treatment doses increased (*n* = 6, F = 29.19, *p* < 0.001, Tukey’s, *p* < 0.05; Figure 4A). While a 24 h exposure to NaAsO_2_ reduced metabolism, NaAsO_2_ treatment induced a non-monotonic dose response with increased proliferation at 0.1 µM, no effect at 1 µM, and decreased proliferation at higher doses compared to the control (*n* = 6, F = 131.9, *p* < 0.001; Tukey’s, *p* < 0.05; Figure 4B).

### 2.4. Exposure to NaAsO_2_ for 72 h Reduces Cell Metabolism and Alters DNA Quantity as a Measure of Viability

Fibroblasts exposed to NaAsO_2_ for 72 h displayed changes in cell metabolism measured by PrestoBlue at all treatment doses (*n* = 10, F = 2040.7, *p* < 0.001; Figure 4D; Tukey’s, *p* < 0.05). There was a dose-dependent decrease in viability with increasing NaAsO_2_ concentrations beginning at 1 µM (Tukey’s, *p* < 0.05; Figure 4D). However, there was an increase in viability in cells exposed to 0.1 µM NaAsO_2_ (Tukey’s, *p* < 0.05; Figure 4D).

Fibroblasts exposed to NaAsO_2_ for 72 h displayed changes in proliferation measured by CyQuant (*n* = 10, F = 2735.1, *p* < 0.001; Figure 4E). This exposure to NaAsO_2_ for 72 h resulted in a dose-dependent decrease in proliferation with increasing NaAsO_2_ concentrations beginning at 1 µM (Tukey’s, *p* < 0.05). Furthermore, increasing time of exposure from 24 to 72 h decreased the LOEL from 2.5 to 1 µM (Figure 4E).

### 2.5. Reduction in Metabolic Output Relative to Viable DNA Content after 24 and 72 h Exposure to NaAsO_2_

A non-monotonic dose-response was observed in which 24 h NaAsO_2_ exposures decreased cellular metabolic rate relative to viable DNA content at all treatment doses compared to control (*n* = 6, F = 7.58, *p* < 0.001; Tukey’s, *p* < 0.05; Figure 4C). Exposure to NaAsO_2_ for 72 h also affected metabolic output per unit of DNA (*n* = 10, F = 19.6, *p* < 0.001) but only at doses 2.5 µM and greater (Tukey’s, *p* < 0.05; Figure 4F).

### 2.6. Wound Healing Processes in Female Mice Are Negatively Affected by NaAsO_2_-Exposure

**Figure 5:**
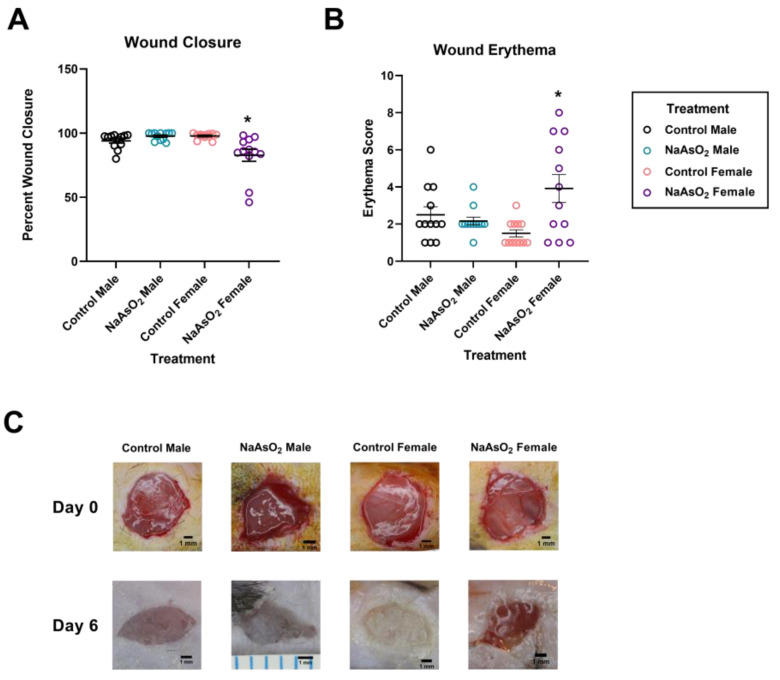
Wound closure areas, wound erythema, and representative wound images at day 6 post-wounding in mice exposed to 10 µM NaAsO_2_ for 8 weeks (**A**) Wound closures in NaAsO_2_-treated females were significantly decreased compared to control females (Chi^2^ = 16.5095, *p* = 0.0022) and NaAsO_2_-treated males (Chi^2^ = 16.5095, *p* = 0.0091). Mean wound closure areas for each treatment group are represented +/− SEM (*n* = 12). (**B**) Erythema scores in NaAsO_2_-treated females were significantly elevated compared to control females (Chi^2^ = 19.3237, *p* < 0.0001). Mean erythema values for each treatment group are represented +/− SEM (*n* = 12). (**C**) Representative gross images of wound sites from vehicle and NaAsO_2_-treated mice on day 0 and on day 6. * Statistically significant values (*p* < 0.05) compared to control (Dunn’s post hoc test).

An 8-week exposure to 10 µM NaAsO_2_ in drinking water impeded wound closure in mice 6 days post-wounding (Chi^2^ = 13.3458; *p* = 0.0039, Figure 5A). Wound closure areas were significantly decreased in NaAsO_2_-treated females compared to control or NaAsO_2_-treated males (Z = 2.817; *p* = 0.0290) and control females (Z = −3.185; *p* = 0.0087; Figure 5A).

NaAsO_2_-treated female mice displayed increased wound erythema levels compared to all other groups (Figure 5B). A Kruskal Wallis test revealed a significant effect among all treatment groups (Chi^2^ = 9.456; *p* = 0.0238). Macroscopic observations revealed increased visual erythema in NaAsO_2_-treated female mice at day 6 with no difference observed in other treatment groups (Figure 5C). Water consumption was significantly higher in males vs. females (ANOVA, *p* < 0.0001) with post hoc tests revealing no difference between control and arsenic male mice (Dunn’s, *p* > 0.9999) and no difference between control and arsenic female mice (Dunn’s, ANOVA *p* = 0.1639).

### 2.7. Altered Gene Expression in Mouse Wound Biopsies due to NaAsO_2_-Exposure

**Figure 6:**
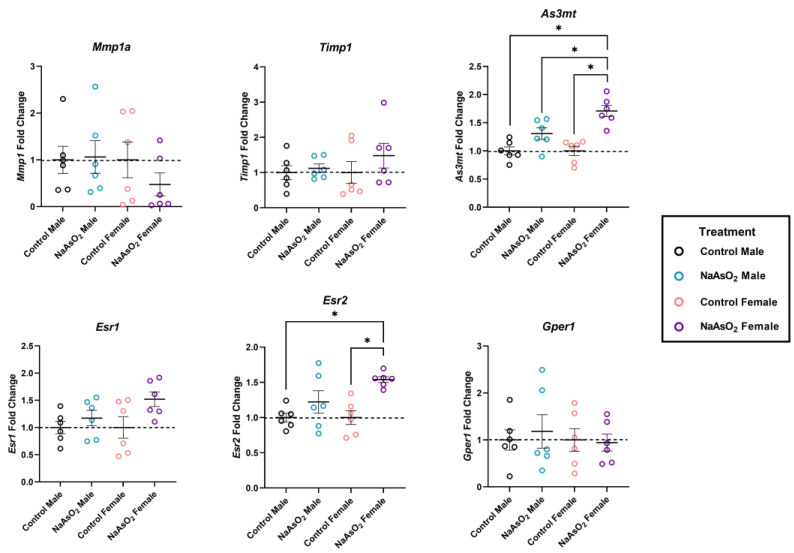
Relative gene expression of arsenic metabolism, matrix remodeling, and estrogen signaling genes in mice exposed to 10 µM NaAsO_2_ for 8 weeks. Exposure to NaAsO_2_ significantly increased *As3mt* expression in NaAsO_2_-treated female mice compared to control groups (W = 12.20, *p* = 0.008). Exposure to NaAsO_2_ significantly increased *Esr2* expression in NaAsO_2_-treated female mice compared to control groups (W = 19.30, *p* = 0.0002). Individual data points represent fold changes per replicate +/− SEM (*n* = 6). * Statistically significant (*p* < 0.05) compared to control (Tukey’s post hoc test).

An 8-week exposure to 10 µM NaAsO_2_ in drinking water altered gene expression in wound biopsies (Figure 6). One-way ANOVAs revealed significant differences in both *As3mt* (W = 12.20; *p* = 0.0008) and *Esr2* expression (W = 19.30; *p* = 0.0002) across groups. *As3mt* was significantly upregulated in wounds of NaAsO_2_-treated females compared to control females, control males, and NaAsO_2_-treated males (*n* = 6; *p* < 0.0001; *p* < 0.0001; *p* = 0.0230, respectively). *Esr2* was significantly upregulated in wounds of NaAsO_2_-treated females compared to control female and NaAsO_2_-treated male groups (*n* = 6; *p* = 0.006; *p* = 0.006, respectively). No significant differences in transcript levels were detected for *Esr1*, *Gper1*, *Mmp1a*, or *Timp1* in wound sites (Figure 6).

## 3. Discussion

In the current study, arsenic (NaAsO_2_) exposure interfered with normal wound healing processes modeled using in vitro and in vivo experimental tests. In cell culture experiments, dermal fibroblasts exposed to environmentally relevant concentrations of NaAsO_2_ displayed slowed scratch closure, decreased cell proliferation, and decreased cell metabolism relative to viable DNA content. Increased transcription of *MMP1* was detected in dermal fibroblasts exposed to NaAsO_2_. In mice, 10 µM NaAsO_2_ negatively impacted wound healing outcomes in female mice displaying slower wound closure and higher erythema scores. Increased transcription of *Esr2* and *As3mt* were also measured in mouse wound biopsies. Here, we demonstrated the use of in vitro techniques to help guide study designs in an in vivo full thickness wound model where NaAsO_2_ exposure was found to impair wound healing functions and outcomes in both models.

Our results demonstrated that scratch closure, proliferation, metabolic activity, and cell viability of fibroblasts were detrimentally affected when exposed to environmentally relevant NaAsO_2_ concentrations. Altered fibroblast activity during the wound healing process may lead to poor wound healing outcomes, including an increased risk for non-healing wounds and infection [32,33]. In the scratch assay, NaAsO_2_ exposure at the time of scratching negatively impacted closure, and 72 h exposure to NaAsO2 prior to scratching exacerbated the effect, in some cases reducing the LOEL by 10-fold. This shift in exposure timing and LOEL suggests that long-term exposure to arsenic is more detrimental to cellular processes than short-term exposure.

Cellular proliferation was also impacted by NaAsO_2_ exposure, which was evident by the dose-dependent decline in cell counts. This trend was consistent with Xiong et al.’s findings of arsenic induced cell cycle arrest in neuroblastoma cells. This group treated cells with similar concentrations of As_2_O_3_ (1, 2, or 4 µM), in which 4 µM induced cell cycle arrest leading to decreased cellular proliferation [34].

The decrease in scratch closure and proliferation of continuously exposed fibroblasts were accompanied by decreased cellular metabolism relative to viable DNA content. The detrimental effects of NaAsO_2_ on metabolic activity and cell viability reported here were expected due to arsenic’s known affinity for thiol groups and disruption of sulfhydryl group-containing enzymes, many of which have prominent roles in several metabolic pathways [35]. In addition to decreases in cell metabolism, increased cell death due to NaAsO_2_ exposure has been previously described. One study found that exposure to low-dose NaAsO_2_ (0.5 µM) exhibited cytotoxic effects both acutely and chronically in lung fibroblasts [36], and our dermal fibroblast viability results were consistent with their studies. Interestingly, 0.1 µM NaAsO_2_ slightly increased proliferation and metabolism in our study. Low levels of arsenic may induce these changes through a variety of pathways, which may include a non-detectable change in oxidative stress and reduced cell autophagy [37]. Like most toxicants, effects of arsenic are likely concentration-dependent, in which increasing concentrations no longer provide stimulatory changes, but rather detrimental consequences [38].

While other matrix remodeling genes were analyzed in this study (not published), only *MMP1* was significantly upregulated in dermal fibroblasts. *MMP1* transcript levels were enriched in NaAsO_2_-treated fibroblasts, which was consistent with a reported significant upregulation of *MMP1* gene expression, however, in human uroepithelial cells (SV-HUC-1) exposed to arsenic [39]. MMP1 functions to remodel the extracellular matrix (ECM) of a healing wound through proteolytic digestion of collagen, which then stimulates fibroblasts to lay down new collagen for cell adherence and subsequent cellularization [40,41]. The dynamic process of ECM remodeling by dermal fibroblasts during tissue remodeling is a delicate balance of collagen deposition and MMP1 enzymatic digestion. For example, increased collagen deposition during the final stages of wound healing can lead to hypertrophic scarring or the formation of a keloid scar [42]. In contrast, elevated levels of MMP1 are implicated in the pathogenesis of chronic wounds; therefore, results from the current study suggest that arsenic may play a role in the pathogenesis of chronic wound formation in individuals who are repeatedly exposed for extended periods [43,44].

While NaAsO_2_ impeded fibroblast functions, multiple cell types, including epithelial cells, fibroblasts, and leukocytes, work together to close and remodel wounds in a living organism. The effects of NaAsO_2_ were further explored in a full thickness wound model in mice to evaluate if in vitro findings translated to in vivo models. Exposure to 10 µM (10 ppb) NaAsO_2_ for 8 weeks diminished wound closure and increased erythema in female mice, with no visual detriments observed in male mice. Sex differences were seen in wound healing for both gross evaluations and gene expression analysis, which suggests disrupted hormone signaling. Additionally, water consumption in our study was significantly different between male and female mice, but not within treatment groups. Sex difference results were expected and have been reported in the literature previously [45]. Water consumption data in our study may have been influenced by a variety of variables including cage movement and water leakage during water changes.

Hormone signaling plays an imperative role in cell communication, specifically in wound healing. Under normal inflammatory processes, neutrophils are rapidly recruited to the wound site in a spatial pattern surrounding the wound [46] and undergo apoptosis after wound bed clearance [47], which slows the recruitment of additional neutrophils. Estrogen inhibits excessive neutrophil recruitment by upregulating mitogen-activated protein kinase phosphatase 2 (MKP-2) [29], and by reducing leukocyte interleukin-1β (IL-1β) secretion [29,48,49]. Because of arsenic’s affinity for endogenous estrogen [30], the presence of NaAsO_2_ in our study may have inhibited estrogen’s ability to promote normal wound healing in female mice.

There is evidence that arsenic acts as an endocrine disruptor through several potential molecular mechanisms. Arsenic binds to estrogen directly [30], and many studies have investigated the endocrine disrupting properties of arsenic [50,51,52,53]; however, there is limited literature regarding arsenic–estrogen interactions in skin. In the current study, we evaluated estrogen receptor mRNA expression in vivo (*Esr1*, *Esr2*, and *Gpr30*) and found that *Esr2* was upregulated in the wound sites of female mice exposed to NaAsO2. In skin, *Esr1* exhibits low expression [54], while *Esr2* expression is widespread [55]. One study showed that knockout mice for estrogen receptor beta (Erβ), encoded by the Esr2 gene, showed impaired wound healing [56], indicating that Erβ activity in the skin is vital to wound closure. These results suggest that arsenic may interfere with wound healing through disruption of estrogen signaling.

There was a significant upregulation of the *As3mt* gene in the wound sites of both sexes in vivo. Arsenic methyltransferase (*AS3MT*) detoxifies inorganic arsenic in many species including humans [57], rodents [58], and fish [59,60]. The upregulation of the *AS3MT* gene in the skin of both male and female mice exposed to NaAsO_2_ in this study demonstrated the ability of NaAsO_2_ to affect the skin. One study found that *As3mt* is required for NaAsO_2_ metabolism in mice, with *As3mt* knockout mice retaining toxic arsenic metabolites in tissues [61]. Interestingly, polymorphisms in the human *AS3MT* gene may also lead to a higher risk of arsenic-induced premalignant skin lesions [62], indicating that human AS3MT expression in skin may play a protective role. The *As3mt* upregulation in the wound sites of mice in the current study may have been a sign of detoxification in the skin, and therefore may have been protective. Additionally, one study has previously shown that when exposed to arsenate, GAPDH acts as an AsV reductase, and therefore shows that in the presence of arsenate and other isoforms of arsenic, GAPDH is present [63]. The presence of GAPDH during arsenic detoxification supports the use of this gene as a housekeeping gene in these studies.

In normal human wound healing processes, MMP1 degrades type 1 collagen to remodel the wound bed, while its inhibitor, TIMP1, acts to inhibit MMP1 activity [64,65]. The in vivo results paralleled the cytotoxic effects seen in fibroblasts exposed to 10 µM NaAsO_2_ for 72 h. While there were no significant differences in the expression of *Mmp1a* and Timp1, the expression of other collagenases, gelatinases, and MMP inhibitors may have changed in response to NaAsO_2_ exposure for 72 h. In our study, wound sites may have had reduced ECM at the time of tissue collection (6 days post-wound creation). Another study identified a 7–14 day matrix remodeling phase in murine wound models [66]. This finding of a longer murine wound remodeling phase may suggest that the timepoint of tissue collection was critical for analysis of matrix remodeling genes.

Limitations to our study include time of tissue collection, which is mentioned previously, and length of arsenic exposure. Some studies utilizing a chronic model of arsenic exposure dosed for more than 8 weeks [66]; however, longer exposure times were out of the scope of the current study. Additionally, dermal protein expression, histological analysis, and flow cytometry may provide more insight into cellular changes; however, these studies are out of the scope of the current study.

These data underscore the significance to human and environmental health concerns of long-term arsenic exposure. This study used NaAsO_2_ concentrations between 0.1 and 10 µM, which capture the USEPA MCL of 0.13 µM (10 ppb); however, global surface and groundwater resources often contain levels well above 0.13 µM. These arsenic-contaminated resources (many above 10 µM) are found globally, including areas in Asia and the Americas [1,4,5,12,67,68,69,70]. Overall, these data suggest that individuals who experience delayed wound closure and/or the formation of chronic non-healing skin wounds are at particular risk if chronically exposed to arsenic.

## 4. Materials and Methods

### 4.1. Arsenic Preparation

Sodium arsenite (CAS#: 7784-46-5, Sigma-Aldrich, St. Louis, MO, USA) was weighed and diluted in reverse osmosis deionized (DI) water to create a 10 mM stock solution. For cell culture assays and mouse experiments, 10 mM NaAsO_2_ stocks were further diluted into either cell culture medium or reconditioned RO water to reach a final concentration of 10 µM.

### 4.2. General Cell Culture Conditions

Human neonatal dermal fibroblasts (hDFn), primary isolates up to 5th passage, (Cell Applications, Inc, San Diego, CA, USA) were grown as a monolayer in a tissue culture treated T75 flask (Corning Inc., Corning, NY, USA) at a seeding density of 5000 cells/cm^2^ and passaged at 70–80% confluence. All cell counts were generated using a hemocytometer. The cells were grown in Dulbecco’s Modified Eagle Medium (DMEM, Life Technologies, Carlsbad, CA, USA) supplemented with 10% *v*/*v* fetal bovine serum (FBS, Life Technologies, Carlsbad, CA, USA). Cells were incubated at 37 °C, 5% CO_2_.

### 4.3. Exposure for 24 h and 72 h Cell Scratch Assay

Cells were sub-cultured into a tissue culture-treated 12-well plate (Corning Inc., Corning, NY, USA) at a seeding density of 5000 cells/cm^2^ and incubated for 2–3 days until the cells reached 100% confluence. Upon reaching confluence, the monolayer was scratched by hand using a P200 pipet tip (Gilson Inc., Middleton, WI, USA). Next, the media were removed, the wells rinsed with 1x Hanks Balanced Salt Solution (HBSS, Life Technologies, Carlsbad, CA, USA) to remove detached cells, and NaAsO_2_ in DMEM at 0.01, 0.1, 0.5, 1, and 10 µM concentrations were added. Independent scratch assays were conducted to assess the effect of 24 h versus 72 h NaAsO_2_ exposure on wound closure. In the 24 h exposure assays, the cells were scratched and concomitantly exposed to NaAsO2 concentrations. In the 72 h exposure assays, cells were exposed to NaAsO_2_ for 72 h prior to performing the scratch. In both assays, an inverted microscope and digital camera (Leica, Wetzlar, Germany) were used to capture images every four hours for 24 h to record cellular migration. Scratch closure was quantified for all experimental conditions and compared to control conditions using an automated algorithm that was developed to quantify cellular migration using MatLab (See Supplemental File). Summed area under the curve (AUC) was calculated using the summed trapezoid method of the percent closure values over a 24 h period from individual wells. At the experiment’s conclusion, all cells were lysed in 300 µL TRIzol reagent (Thermo Fisher, Waltham, MA, USA), and the lysates were stored at −80 °C to be used later for gene expression analysis.

### 4.4. Quantitative Polymerase Chain Reaction (qPCR) in Human Dermal Fibroblasts

Total RNA was isolated from the same cells used in the scratch assay (stored in TRIzol at −80 °C) with a DirectZol MicroPrep RNA kit (Zymo, Irvine, CA, USA). RNA quantity and ribosomal RNA integrity (RIN) was determined using a fragment analyzer (Advanced Analytical, Ankeny, IA, USA). First-strand cDNA was synthesized using the iScript cDNA synthesis kit (BioRad, Hercules, CA, USA) from 1 µg of RNA. All qPCR assays utilized primers designed using design software (Primer3, NCBI: https://www.ncbi.nlm.nih.gov/tools/primer-blast/ accessed 1 August 2019) and synthesized by Integrated DNA Technologies (Coralville, IA, USA) (Table 1). A standard curve was run for each primer set, and melting peaks were assessed. All assays were performed using qPCR SYBR Green Master Mix chemistry (BioRad, Hercules, CA, USA) and a CFX384-TOUCH real-time thermocycler. All samples were conducted in quadruplicate technical replicates. Cycling conditions were polymerase activation at 95 °C (30 s), followed by 40 amplification cycles of template denaturation at 95 °C (15 s), primer annealing/extension/detection at 60 °C (30 s), and a melt analysis at 65–95 °C (Δ0.5 °C/5 s). Expression was determined using the delta-delta cT method and presented as “log 2-fold change” relative to untreated control samples normalized to the geometric mean of 2 housekeeping genes in control and treated samples.

### 4.5. Cell Proliferation and Viability Assays

Human neonatal dermal fibroblasts (hDFn) were grown as a monolayer in a tissue culture treated T75 flask at a seeding density of 5000 cells/cm^2^ and passaged at 70–80% confluence. Cells were sub-cultured into a black wall, clear bottom, tissue culture-treated 96-well plate (Corning Inc., Corning, NY, USA) at a seeding density of 7000 cells/cm^2^ in 200 µL media/well, with 200 µL water blanks in outer periphery of wells to prevent media evaporation of inner treatment wells. Cells were grown for 24 h, then exposed to NaAsO_2_ concentrations (0, 0.1, 1, 2.5, 5, and 10 µM) for either 24 or 72 h prior to the addition of fluorescent dyes. On the day of experiment, PrestoBlue (PB, Invitrogen, Carlsbad, CA, USA) and CyQuant (CQ, Invitrogen, Carlsbad, CA, USA) solutions were prepared according to the manufacturer’s protocol. A portion of media was removed from each of the wells (150 µL) and replaced with 50 µL diluted PB, leaving 100 µL total volume per well. Once PB was added, the plate was incubated for 10 min at 37 °C, 5% CO_2_ and read on a Biotek Synergy HT fluorescent plate reader (Biotek, Winooski, VT, USA) at 560/590 nm and gain set at 45. Next, 100 µL of CQ diluent was added to each well. The plate was incubated for 60 min at 37 °C, 5% CO_2_ and read on a Biotek Synergy HT fluorescent plate reader at 480/538 nm and gain set at 70. Relative fluorescent units (RFU) were generated for each well, which was directly correlated to cell metabolic output per unit DNA.

### 4.6. Growth Curve Assay

Cells were sub-cultured into tissue culture-treated 12-well plates at a seeding density of 2630 cells/cm^2^ into a total of 12 plates. At the time of seeding in 12-well plates, concentrations of NaAsO_2_ (0, 0.1, 0.5, 1, and 10 µM) were added. Cells from 4 wells/plate were counted using a hemocytometer every 24 h over a 6-day period. At the end of the 6-day period, the cell counts from each day were used to assess fibroblast proliferation in the presence of increasing NaAsO_2_.

### 4.7. Full-Thickness Wound Creation and Image Analysis

Twelve female and twelve male 8-week-old mice (Mus musculus C57BL/6) were purchased from Jackson Laboratory (Bar Harbor, ME, USA). These mice were used in accordance with Northern Arizona University’s (NAU) Institutional Animal Care and Use Committee (Protocol# 18-018). The mice arrived in a healthy condition at NAU’s Research Annex and were allowed a 28-day acclimatization period, during which animal care staff placed mice in cages in a blinded fashion without interference from research staff. Mice were randomly selected (6 female and 6 male) to receive RO water containing 10 µM NaAsO_2_, while the rest of the groups (6 female and 6 male) received RO water. Once dosing began, cages and food were changed weekly, and water was changed every 3 days. Water intake was measured per cage every 3 days using a 500 mL graduated cylinder.

After 8 weeks of dosing, mice were anesthetized using 3% Isoflurane and two full-thickness wounds were created, one on each side of the midline of the dorsum of each animal using a sterile 6 mm dermal punch and tissue excision following prior methods [71]. All animals were treated with Tegaderm (3M., St Paul, MN, USA) dressings to cover the wound sites and butterfly harnesses (LOMIR INC., Malone, NY, USA) to prevent animal interference with wound sites. Mice were individually housed for the duration of the experiment. Left- and right-wound sites for each mouse were photographed with a Nikon DSLR camera with lightbox and macro lens attachments on days 0 and 6.

Wound images from days 0 and 6 post-operation were analyzed with ImageJ software v.1.54h (U. S. National Institutes of Health, Bethesda, MD, USA). Scale was determined by measuring a 1 mm line on the ruler in every photo (using the line tool). Wound areas were traced along the inside wound edge (using the freehand selections tool) and the total area was computed. Percent wound closure was calculated using the below equation:

% Wound Closure = [(Initial Wound Area − Final Wound Area) × 100]/(Initial Wound Area)

Erythema was measured using a qualitative rating scale for all images at each timepoint across cohorts. Images were ranked from 1–10, with 1 being the least red and 10 being the reddest.

### 4.8. Tissue Collection and Quantitative Polymerase Chain Reaction (qPCR) in Mouse Wound Biopsies

On day 6, all animals were euthanized by 5% Isoflurane overdose and cervical dislocation. Animals were weighed and all wound areas (2 per mouse) were excised, placed in 1 mL RNAlater™ Stabilization Solution (Thermo Fisher, Waltham, WA, USA), and stored at −80 °C until RNA isolation. Dermal punches were sent to University of Arizona Genetics Core (Tucson, AZ, USA) for RNA isolation. Total RNA was isolated from each mouse skin tissue sample using the Invitrogen PureLink RNA Mini extraction kit (Invitrogen, Carlsbad, CA, USA). RNA quantity and ribosomal RNA integrity (RIN) was determined using the 2100 Bioanalyzer Instrument (Agilent Technologies, Santa Clara, CA, USA). Primers for *Gapdh*, *Sdha*, *As3mt*, *Mmp1a*, *Timp1*, *Esr1*, *Esr2*, and *Gper1* were designed using the total gene sequences (Primer3, NCBI) for each target gene assessed (Integrated Data Technologies, Coralville, IA, USA) (Table 1). A standard curve was run for each primer set and melting peaks were assessed. Reagents for cDNA synthesis/qPCR and thermocycler settings were previously described.

### 4.9. Statistical Analysis

Statistically significant differences among experimental conditions in vitro were determined using the R-Program for Statistical Computation (V3.2.2 GUI 2014) using an ANOVA with a Tukey’s HSD post hoc evaluation. In vivo data were statistically evaluated using JMP^®^ (Version 15. SAS Institute Inc., Cary, NC, USA) software. Data were evaluated for normality and homogeneity of variance, with the alpha set at 0.05. Wound area, erythema endpoints, and water consumption were assessed using a one-way Kruskal Wallis test with a Dunn’s post hoc test for non-parametric data. Transcript levels assayed by qPCR were compared using a one-way ANOVA and Tukey’s HSD post hoc evaluation. Graphing was performed using GraphPad Prism version 9.0.0 for Windows, GraphPad Software v9.4.1, San Diego, CA, USA.

## Figures and Tables

**Table 1:** Real-time qPCR gene targets and primer sequences for human dermal fibroblasts and Mus musculus.

Gene Transcript	Gene Abbreviation	Primer Sequence	Annealing Temperature (°C)	Amplicon Length
**Human**				
Glyceraldehyde 3-phosphate dehydrogenase	GAPDH	F-CTCCAAAATCAAGTGGGGCGA	60.89	70
		R-CATGGTGGTGAAGACGCCAG	61.3	
Succinate dehydrogenase complex subunit A	SDHA	F-TGGCCCTGAGAAAGATCACG	59.75	193
		R-GACCTGCCCCTTGTAGTTGG	60.32	
Matrix metalloproteinase 1	MMP1	F-GGCCACAAAGTTGATGCAGTT	59.93	137
		R-TTCCTGCAGTTGAACCAGCTA	59.58	
**Mouse**				
Glyceraldehyde 3-phosphate dehydrogenase	GAPDH	F-CATCACTGCCACCCAGAAGACTG	63.28	153
		R-ATGCCAGTGAGCTTCCCGTTCAG	65.15	
Succinate dehydrogenase complex subunit A	SDHA	F-GAGATACGCACCTGTTGCCAAG	61.82	113
		R-GGTAGACGTGATCTTTCTCAGGG	60.18	
Matrix metalloproteinase 1a	MMP1a	F-AGGAAGGCGATATTGTGCTCTCC	62.37	98
		R-TGGCTGGAAAGTGTGTGAGCAAGC	63.85	
TIMP metallopeptidase inhibitor 1	TIMP1	F-TCTTGGTTCCCTGGCGTACTCT	63.08	131
		R-GTGAGTGTCACTCTCCAGTTTGC	61.59	
Arsenic 3-methyltransferase	AS3MT	F-TCCACGTTTGGTCACTGCCGAT	64.71	100
		R-GAAGAGGCGAAATGTGGCAGAC	62.07	
G-Protein coupled estrogen receptor 1	GPER1	F-GCCACATAGTCAACCTTGCAGC	62.34	113
		R-CGTCTTCTGCTCCACATAGAGC	60.8	
Estrogen receptor 1	ESR1	F-TCTGCCAAGGAGACTCGCTACT	62.86	153
		R-GGTGCATTGGTTTGTAGCTGGAC	62.46	
Estrogen receptor 2	ESR2	F-GGTCCTGTGAAGGATGTAAGGC	60.68	139
		R-TAACACTTGCGAAGTCGGCAGG	63.35	

## Data Availability

The data presented in this study are available on request from the corresponding author. The data are not publicly available due to confidentiality concerns.

## Supplementary Materials

The following supporting information can be downloaded at: https://www.mdpi.com/article/10.3390/ijms25042161/s1.
